# The Molecular Weight of Enzymatically Modified Pectic Oligosaccharides from Apple Pomace as a Determinant for Biological and Prebiotic Activity

**DOI:** 10.3390/molecules30010046

**Published:** 2024-12-26

**Authors:** Agnieszka Wilkowska, Adriana Nowak, Ilona Motyl, Joanna Oracz

**Affiliations:** 1 Institute of Fermentation Technology and Microbiology, Faculty of Biotechnology and Food Sciences, Łódź University of Technology, Wólczańska 171/173, 90-530 Łódź, Poland; 2 Department of Environmental Biotechnology, Faculty of Biotechnology and Food Sciences, Lodz University of Technology, Wólczańska 171/173, 90-530 Łódź, Poland; adriana.nowak@p.lodz.pl (A.N.); ilona.motyl@p.lodz.pl (I.M.); 3 Institute of Food Technology and Analysis, Faculty of Biotechnology and Food Sciences, Lodz University of Technology, B. Stefanowskiego 2/22, 90-537 Łódź, Poland; joanna.oracz@p.lodz.pl

**Keywords:** pectin derived oligosaccharides (POSs), apple pomace, prebiotics, molecular weight

## Abstract

The purpose of this research was to investigate the prebiotic effects of different fractions of pectin-derived oligosaccharides (POSs) from apple pomace (AP) in relation to their molecular weight (MW), structure, and composition. Enzymatic treatment of the apple pomace resulted in high-molecular-weight arabinans and rhamnogalacturonans (MW 30–100 kDa, MW 10–30 kDa), as well as oligomeric fractions with molecular weights of less than 10 kDa, consisting mainly of homogalacturonan. The biological potential of the POSs against various lactobacilli and bifidobacteria was evaluated. The oligosaccharides with the highest molecular weights (MW 30–100 kDa, MW 10–30 kDa) showed better prebiotic effect to lactobacilli. The oligosaccharides with MW 3–10 kDa and MW 10–30 kDa caused an increase in the bifidogenic effect. Inhibition of *Escherichia coli*, *Salmonella enterica* serovar Typhimurium, and *Listeria monocytogenes* was also observed. The preparations with MW 3–10 kDa and MW 10–30 kDa demonstrated the strongest biological activity, supporting the adhesion of beneficial microorganisms to mucin and collagen surfaces. Therefore, oligosaccharides with MW 10–30 kDa were considered to be the most promising prebiotic candidates. This study confirms that the biological effects of pectic oligosaccharides vary significantly based on their structural differences. Therefore, the conditions of enzymatic hydrolysis of apple pectin should be optimized to obtain oligosaccharides within a specific molecular mass range.

## 1. Introduction

Pectin is a naturally occurring plant cell wall biopolymer with an expanding range of pharmaceutical and biotechnological applications, especially in health promotion and therapeutic treatments. Due to its gel-forming properties, pectin is also used in the food industry as a thickening agent and colloidal stabilizer. Pectin molecules contain from a few hundred to around one thousand monosaccharide units, corresponding to average molecular weights of about 50–150 kDa. Their composition, structure, and molecular weight depend on factors such as the source (e.g., fruits, vegetables), the degree of maturity of the plant or fruit, and the extraction methods used. Due to such high complexity and variability, the exact structure of pectin has still not been definitively established. The molecular weight and the content of particular subunits may differ in any given sample of pectin from molecule to molecule [1,2,3]. According to recent findings, pectin heteropolysaccharide is composed of three main structural domains: homogalacturonan HG (consisting of a backbone of α-1,4-linked residues of galacturonic acid) and two types of highly branched rhamnogalacturonans, designated as RG-I (branched with a single-unit β-D-galactose as well as polymeric substitutions, such as arabinogalactan I (AG I) or arabinan) and RG-II (containing side chains with very peculiar sugar residues, such as apiose, aceric acid, 3-deoxy-lyxo-2-heptulosaric acid, or 3-deoxy-manno-2-octulosonic acid) [4,5,6].

Recently, there has been increasing interest in pectins as a valuable source of prebiotic oligosaccharides. Prebiotics are selectively fermented ingredients that promote specific gastrointestinal microbiota. By promoting the growth of these beneficial microbes, prebiotics can help improve gut health and overall well-being [7]. Additional health-promoting effects include the generation of short-chain fatty acids (SCFAs) [8], immunomodulating activity [9], antagonism of galectin-3 [10], ameliorating hepatic inflammation [11], antiadhesive activity against gastrointestinal pathogens [12,13], and protection of colonic cells against Siha toxins [14].

Pectinolytic enzymes are powerful tools that can modify the structure of oligosaccharides derived from pectins. These enzymes are highly specific in their activity, due to their preferences for particular substrates, mechanisms of cleavage, and the types of splitting glycosidic bonds they target. The beneficial prebiotic effects of pectin-derived oligosaccharides depend on their chemical and physical characteristics [15]. Islamova et al. [16] evaluated the prebiotic activity of different pectin polysaccharides, concluding that the source of pectin has a significant effect on gut microbiota. According to Holck et al. [17], long-chain arabino-oligosaccharides (DP 7–14) can have a larger bifidogenic effect than short-chain arabino-oligosaccharides (DP 2–10). Sulek et al. [18] compared the prebiotic effects of various high-mass (HA, >1 kDa) and very low-mass (LA, <1 kDa) sugar beet arabino-oligosaccharides. Recent research studied the composition of POS fractions as a function of the method of pectin hydrolysis and their prebiotic index. Different fractionation methodologies were applied. However, little is known concerning the impact of the molecular weight of POS fractions on their ability to support the adherence of beneficial microorganisms to epithelial colon cells. Collagen and the mucous layer play important roles in the adherence of microorganisms to human epithelial colon cells, contributing to colonization and infection. Strains attach to epithelial cells, the mucous layer, and/or extracellular matrices, which is crucial for their survival and the colonization of the host organism [19]. The lining of the intestine is composed of various cell types, including goblet cells that produce glycoproteins, called mucins. These molecules polymerize together with glycolipids to form a physical barrier that protects against pathogens, enzymes, toxins, dehydration, and abrasion [20]. The layer of mucous also provides a place where microbiota can adhere and a nutritive source for commensal microorganisms that promote their growth [21]. The composition of the extracellular matrix surrounding epithelial colon cells is composed of a vast range of components, such as laminin, collagen, and fibronectin, which have been found to be targets for bacterial adherence and colonization/infection [19,22]. According to the literature, a collagen-binding protein (Cbp) from *Lactobacillus* species is responsible for binding to human collagen proteins, and may have a crucial role in gut colonization and the displacement of pathogens [23]. More work is needed to understand the structure and composition of the POS fractions characterized by different molecular weights on their prebiotic index as well as the ability to support the adherence of beneficial and pathogenic microorganisms to epithelial colon cells. This understanding would help to improve the process of obtaining large amounts of highly active prebiotics.

The purpose of this research was to determine how the prebiotic effect of apple pectin oligosaccharides with different molecular weights vary depending on their contents of HG and RG. We investigated whether in vitro fermentation of differently sized POS molecules produced varying effects on selected beneficial as well as pathogenic bacterial strains of gut microbiota in terms of growth stimulation or inhibition as well as their ability to adhere to mucous and collagen surfaces.

## 2. Results and Discussion

### 2.1. Structural Characterization of POSs

The identification and quantification of the structural units of POSs is difficult, due to the complex chemical composition of pectin. Characterization of the POS structure requires a combination of several purification and separation steps, as well as advanced analytical techniques (e.g., NMR, MALDI-TOF) coupled with hydrolysis methods.

In this study, oligosaccharide fractionation was performed by ultrafiltration. The initial extract was fractionated using a 100 kDa, 30 kDa, and 10 kDa molecular weight cut-off (MWCO) ultrafiltration membrane. Three oligosaccharide fractions were obtained:

F 00—oligosaccharide mass in the range of 30–100 kDa (MW 30–100 kDa);
F 01—oligosaccharide mass in the range of 10–30 kDa (MW 10–30 kDa);
F 02—oligosaccharide mass < 10 kDa (MW < 10 kDa).

**Figure 1:**
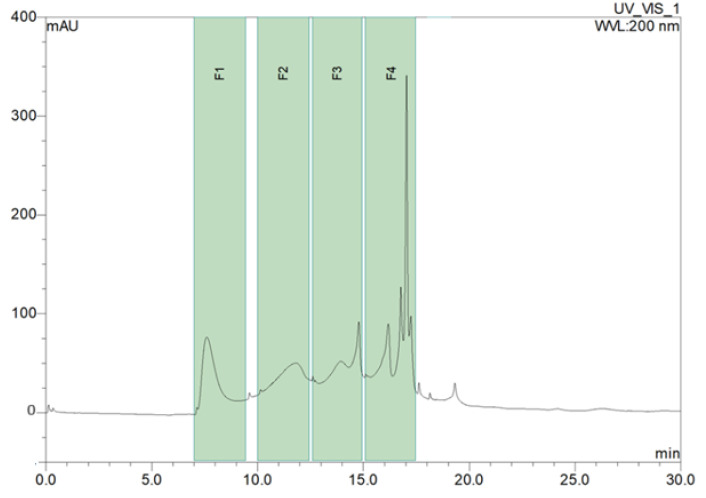
HPSEC separation of the F02 AP hydrolysate fraction (Mw < 10 kDa).

The molecular weights of pectins vary depending on the starting material and the extraction conditions. The relative molecular mass of apple pectin is approximately 122–280 kDa [24,25]. Therefore, 100 kDa and 30 kDa membranes were used to remove large polysaccharide molecules from the AP hydrolysate. Portions of the resulting F 02 fraction (MW < 10 kDa) were further fractionated using high-performance size exclusion chromatography (HPSEC). Figure 1 shows the molecular weight distributions of the collected fractions. Four pools were obtained: F1 (MW 3–10 kDa), F2 (MW 1–3 kDa), F3, and F4 (MW 100 Da–1 kDa).

**Figure 2:**
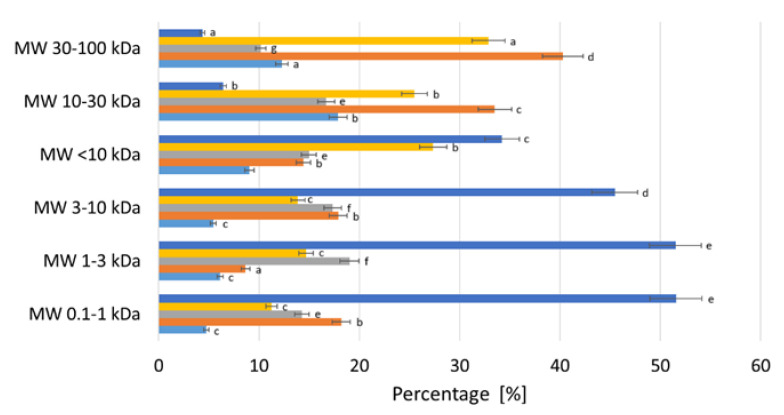
Monosaccharide compositions [%] for POS fractions with different molecular masses. All values are mean ± standard deviation (SD) for triplicate experiments. Values denoted by different letters indicate statistically significant differences between the different MW samples within the same monosaccharide type, ANOVA (*p* < 0.05).

To estimate the oligomeric units, the ratios of various monosaccharides produced through acidic hydrolysis were analyzed. The detailed monosaccharide composition is presented in Figure 2. Concerning the monosaccharide profiles, remarkable differences in the contents of neutral sugars and galacturonic acid were observed for POSs of different molecular weights. The high molecular fractions were composed mostly of neutral sugars, which accounted for 95.64% of the MW 30–100 kDa fraction and for 93.6% of the MW 10–30 kDa fraction. The main degradation products were rhamnose (40.3% and 33.5%) and arabinose (32.9% and 25.5%). Xylose and galactose were detected in lower amounts, ranging from 12.3 to 17.9% and from 10.2 to 16.7%, respectively. Galacturonic acid increased considerably in all POS fractions of low MW (below 10 kDa). The highest amount of galacturonic acid at 51.5% was detected in the oligosaccharide fractions MW 0.1–1 kDa and 1–3 kDa. The amount of galacturonic acid in the MW 3–10 kDa fraction was 45.5%. Rhamnose accounted for 14.4% of the fraction with an MW below 10 kDa, in comparison with 40.3% for the MW 30–100 kDa fraction. These findings are in agreement with Mehrlander (2002) [24], who reported that the low molecular fraction of pectin oligosaccharides in apple juice treated with pectinolytic enzymes consisted almost entirely of galacturonic acid.

**Figure 3:**
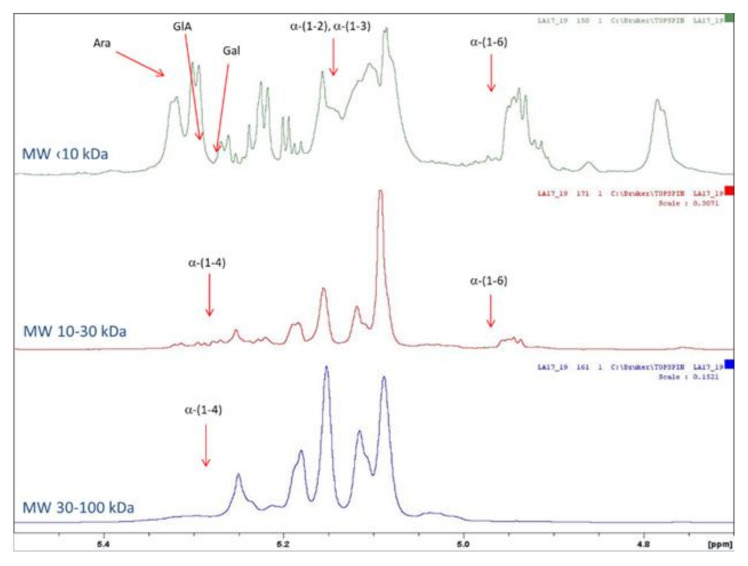
Anomeric region of ^1^H NMR spectrum of POS fractions.

**Figure 4:**
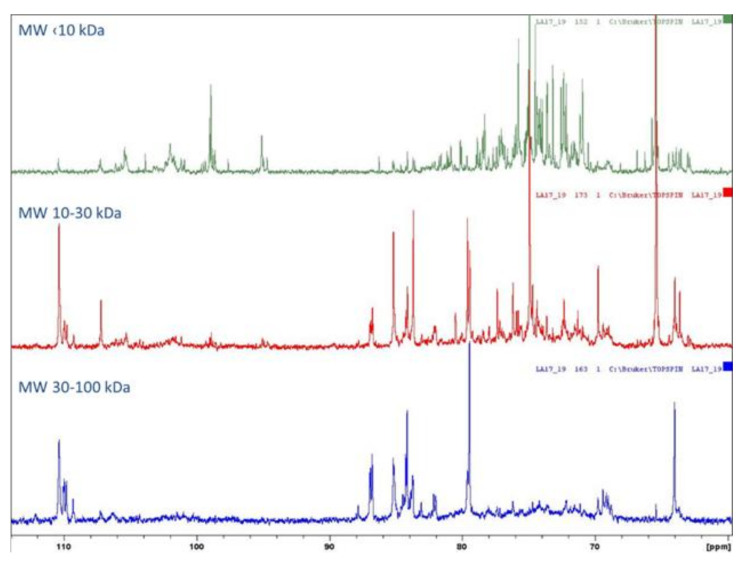
^13^C NMR spectrum of POS fractions.

**Figure 5:**
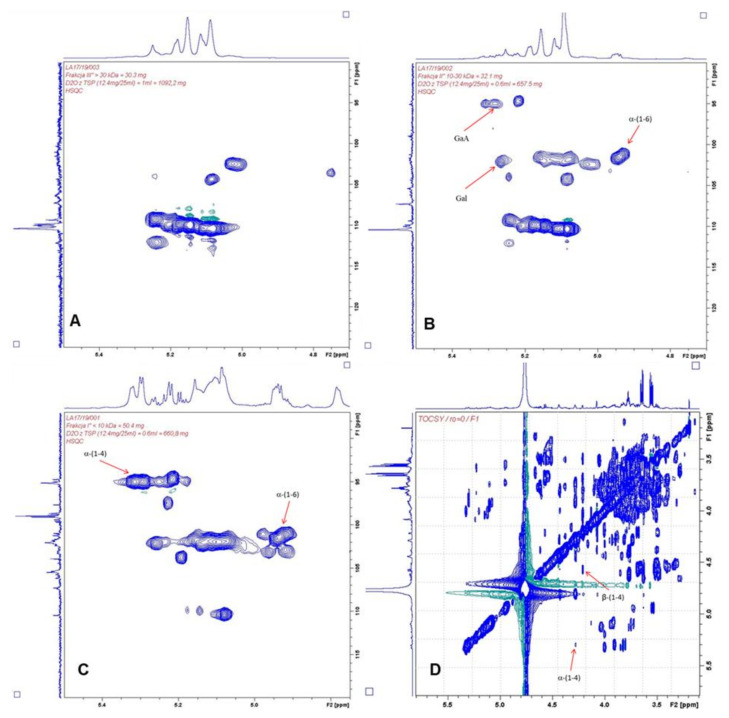
Glucosidic region of HSQC (heteronuclear multiple bond correlation) NMR of POS fractions: (**A**)—MW < 10 kDa; (**B**)—MW 10–30 kDa; (**C**)—MW 30–100 kDa; (**D**)—TOCSY spectrum of MW < 10 kDa fraction.

Holck et al. [26] used a regenerated cellulose membrane of 3 kDa MWCO to purify low-molecular-weight contaminants from POSs, which were intended for use as food ingredients. Iwasaki et al. [27] employed 15 kDa MWCO to wash out sucrose and monosaccharides. In our study, to confirm the structure of the POSs, fractions with MW in the range of 3–100 kDa were subjected to NMR analysis. However, the results were generally difficult to interpret, due to the large numbers of signals in the ^1^H NMR spectrum in the anomeric region. The resolution was too low for reliable structural analyses of individual oligosaccharide molecules. The ^1^H NMR, ^13^C NMR, HSQC, and TOCSY spectra are shown in Figure 3, Figure 4 and Figure 5. For the POS fraction with MW < 10 kDa, the TOCSY correlation spectrum indicated the occurrence of (1–4)-type bonds derived from homogalacturonate (HG). In addition, the sample contained the following sugars: arabinose, rhamnose, xylose, and galactose.

In the 10–30 kDa fraction, the ^1^H NMR spectrum was dominated by two signals with shifts of 3.65 ppm and 3.56 ppm, which we were unable to accurately assign to specific molecular relationships. The same relationship occurred in the MW 3–10 kDa fraction, while in the ^1^H NMR spectrum for the MW 10–30 kDa sample these signals had much lower intensity. This may have been due to the presence of very-high-molecular-weight compounds in this fraction. The fraction of POSs with MW in the range of 30–100 kDa was the most difficult to interpret, due to overlapping signals on the ^1^H NMR spectrum in the anomeric region and the small number of correlation signals on the HSQC spectrum. Due to the presence of very-high-molecular-weight compounds in this sample, we were unable to observe signals from anomeric protons derived from low-molecular-weight saccharides.

According to the FAO, pectin consists of at least 65% galacturonic acid. Sugars can exist in furanic or pyranic forms and with different anomers (α or β), with various types of linkages between monomers, such as α(1-4), α(1-5), β(1-3), β(1-4), and β(1-6) [4,28]. The obtained spectra are generally in good agreement with data obtained after POS acidic hydrolysis. This supports the assumption that the fraction of high molecular weight (MW > 10 kDa) consists of a rhamnogalacturonan core structure, due to the presence of significant amounts of rhamnose, galacturonic acid, arabinose, and galactose. The arabinan backbone was branched in positions 2 and 3. Small amounts of type I arabinogalactans carrying 1,6-linked galactosyl side chains were detected (Table 1). Similar results were reported by Mehländer et al. (2002) [24], who detected mainly α-1,5-linked arabinosyl units branched in positions 2 and 3, as well as 1,4-linked galactosyl with several side chains at positions 2, 3, and 6, representing type I arabinogalactans.

To summarize, the enzymatic treatment of apple pomace resulted in high-molecular-weight arabinans and rhamnogalacturonans as well as oligomeric fractions of molecular weights below 10 kDa, consisting mainly of homogalacturonan.

### 2.2. POS Fermentability

**Figure 6:**
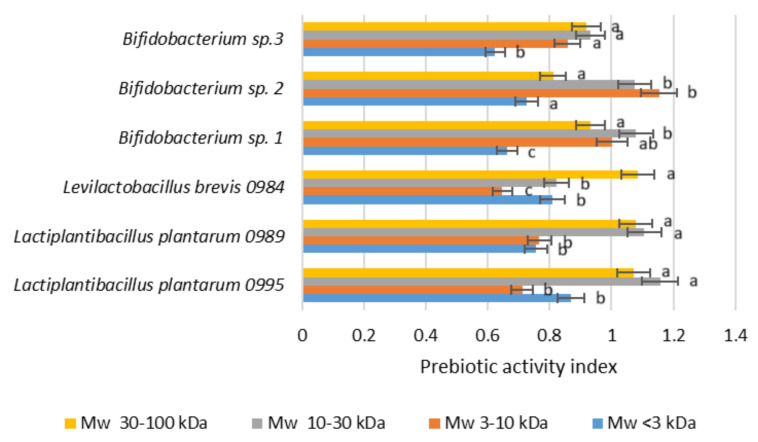
Prebiotic activity index (evaluated by comparison with untreated apple pectin). Data represent means from three replicates in one experiment. Error bars denote SD. Values denoted by different letters indicate statistically significant differences between the oligosaccharide MW samples tested within the same microorganism type, ANOVA (*p* < 0.05).

To investigate the fermentability of POSs of different MW by human intestinal microbiota, 6 commensal and 6 pathogenic bacteria were individually cultured with tested oligosaccharides as a carbohydrate source. The prebiotic potential of high- and low-molecular-weight oligosaccharides was assessed in terms of its ability to support the growth of *Lactiplantibacillus* sp., *Levilactobacillus* sp., and *Bifidobacterium* sp., which are crucial for the proper functioning of the human gastrointestinal tract. The prebiotic potential of POSs was evaluated by comparison with untreated apple pectin. As shown in Figure 6, all tested carbohydrate sources resulted in a significant increase in lactobacilli and *Bifidobacterium* sp. The response to oligosaccharides of the highest molecular weight, MW 30–100 kDa as well as MW 10–30 kDa, was better for lactobacilli, whose growth increased from 1.10- to 1.15-fold in comparison with untreated pectin. Taking into account the growth rate of bifidobacteria, oligosaccharides of MW 3–10 kDa and MW 10–30 kDa were the most promising prebiotic candidates. The growth rate increase recorded for the tested *Bifidobacterium* sp. was in the range of 0.85–1.15 and 0.93–1.08 for MW 3–10 kDa and MW 10–30 kDa, respectively. The lowest values of prebiotic activity indexes were noted for the oligosaccharides of low molecular weight (MW < 3 kDa), ranging from 0.62 to 0.86. This indicates that pectic oligosaccharides with different structures have significantly different biological effects.

A similar effect was observed by Sulek et al. [18], who studied the prebiotic effects of high-mass (>1 kDa) and low-mass (<1 kDa) sugar beet arabino-oligosaccharides. All tested carbohydrates yielded a significant increase in *Bifidobacterium* sp., of between 1.79-fold (HA) and 1.64-fold (FOS). The influence of high-mass arabino-oligosaccharides (>1 kDa) resembled that for FOS more than that seen for mixed and low-mass arabino-oligosaccharides. Different prebiotic activity indexes were obtained by Zhang et al. [29] for citrus peel oligosaccharides of molecular weights in the ranges of 3000–4000 Da and 2000–3000 Da, and lower than 2000 Da. The sample with the lowest MW < 2000 Da showed the highest prebiotic potential, with prebiotic activity scores of 0.41 for *Lactobacillus paracasei* and 0.92 for *Bifidobacterium bifidum*. The preference of *Bifidobacterium* for low-MW substrates in comparison with their respective oligomers has also been confirmed by Gomez et al. [30].

Gullon et al. [31] assessed the suitability of POSs from apple pomace for *Bifidobacterium* sp. and *Bacteroides* sp. metabolism. Glucooligosaccharides were found to be the most suitable carbon source, followed by a mixture of galactooligosaccharides, xylooligosaccharides, and arabinooligosaccharides. Oligogalacturonides were not metabolized by bifidobacteria but were better substrates for *Bacteroides vulgatus*. These findings are in agreement with our research, in which the lowest prebiotic effects were noticed for the oligosaccharide fraction MW < 3 kDa, containing the highest percentage of galacturonic acid (>60%).

**Figure 7:**
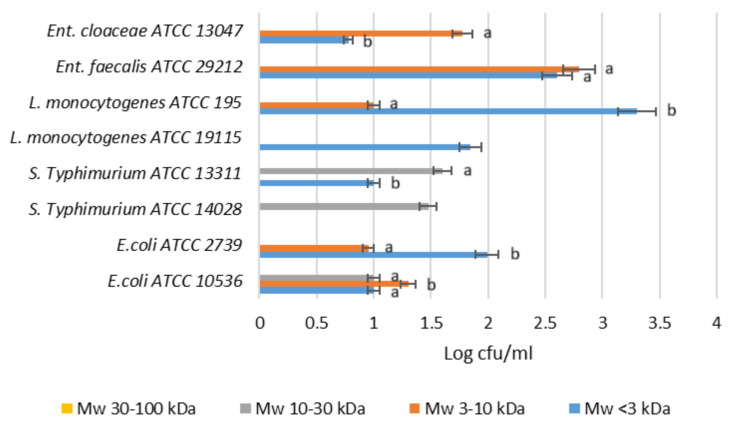
Influence of molecular mass of POSs used as a carbohydrate source on the number of cultured pathogen bacteria. Data represent means from three replicates in one experiment. Error bars denote SD. Values denoted by different letters indicate statistically significant differences between the oligosaccharide MW samples tested within the same microorganism type, ANOVA (*p* < 0.05).

Figure 7 shows the influence of the molecular mass range of POSs used as a carbohydrate source on the number of cultured strains of *Listeria monocytogenes*, *Salmonella enterica* serovar Typhimurium, and *Escherichia coli*. A characteristic property of POSs is their selective fermentability, meaning they should not be fermented by pathogenic microorganisms. The growth of *L. monocytogenes*, *S. Typhimurium*, and *E. coli* was not supported by the high molecular fractions of the apple pomace oligosaccharides, MW 10–30 kDa and MW 30–100 kDa. However, in the case of MW < 3 kDa and MW 3–10 kDa, the growth given as log cfu/mL of the tested pathogens (0.90–1.43) was at a low level. These oligosaccharide fractions may therefore be suitable for use as prebiotics.

### 2.3. Adherence Assay to Collagen and Mucous

**Figure 8:**
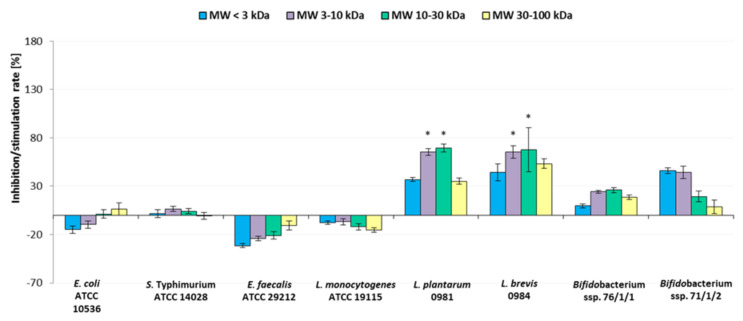
Adherence of bacterial strains to collagen in the presence of apple preparations. Data represent means from four replicates (±SD), where a negative percentage value indicates inhibition of adherence compared to the control sample, and a positive percentage value indicates simulation of adherence compared to the control sample. * Results statistically different from the control (strain in PBS) (ANOVA, *p* < 0.05).

**Figure 9:**
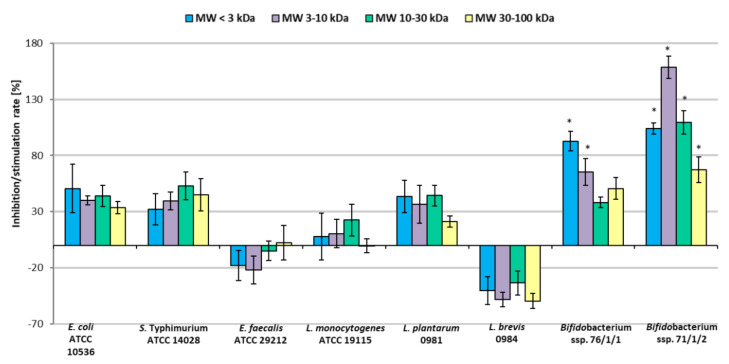
Adherence of bacterial strains to mucous in the presence of apple preparations. Data represent means from four repeats (±SD), where a negative percentage value indicates inhibition of adherence compared to the control sample, and a positive percentage value indicates simulation of adherence compared to the control sample. * Results statistically different from the control (strain in PBS), (ANOVA, *p* < 0.05).

Figure 8 shows the influence of the apple pomace preparations on the adherence of pathogenic and beneficial bacteria to collagen. Figure 9 shows the influence on their adherence to mucous. The results were compared between strains with and without the presence of POSs.

Generally, POS preparation stimulated the adherence of all lactobacilli and *Bifidobacterium* strains to the collagen surface. Stimulation was higher for lactobacilli strains than for *Bifidobacterium*, reaching up to 70% for *Lactiplantibacillus plantarum* ŁOCK 0981 in the case of MW 3–10 kDa and MW 10–30 kDa preparations, which were the most effective (Figure 8) (*p* < 0.05). The stimulation rate for *Bifidobacterium* isolates was weaker, reaching up to 44–46% for the *Bifidobacterium* 71/1/2 strain. The prebiotic preparations had little influence on the adhesion of pathogenic *L. monocytogenes* ATCC 19115, *S. typhimurium* ATCC 14028, and *E. coli* ATCC 10536. Even for *Enterococcus faecalis* ATCC 29212, which showed the highest inhibition, the results were not statistically significant.

On mucous surfaces, the apple preparations stimulated the adherence of *Bifidobacterium* strains by up to 160% in the presence of the prebiotic with MW 3–10 kDa (*p* < 0.05) (Figure 9). Apple pomace oligosaccharides stimulated the adherence of *L. plantarum* ŁOCK 0981 by up to 44% for the 10–30 kDa preparation. Adherence of *Levilactobabillus brevis* ŁOCK 0984 was inhibited by all oligosaccharide preparations. Only in in the case of *E. faecalis* ATCC 29212 was adhesion of pathogenic bacteria inhibited, and in this case only slightly.

Generally, the tested pathogenic bacteria of fecal origin, which are associated with food poisoning, displayed better adherence to the mucous layer than to collagen. The *Bifidobacterium* strains 76/1/1 and 71/1/2 isolated from feces also demonstrated better attraction to mucous than to collagen surface. The presence of the POS preparations also stimulated the adhesive capabilities of these bacteria, to greater or lesser extents.

The effect of POSs contained in the apple pomace hydrolysates on the adhesion of lactic acid bacteria or pathogens to human gut epithelial cells was investigated by Wilkowska et al. [8]. The strongest stimulation of lactic acid bacteria adhesion to the Caco-2 cells occurred in the presence of the hydrolysate containing the highest concentration of higher-order oligosaccharides of polymerization degree DP 7–10. The tested hydrolysates inhibited the pathogens’ adhesion to intestinal cells.

To conclude, the MW 3–10 kDa and MW 10–30 kDa preparations appeared to demonstrate the greatest biological activity towards adhesion of the tested microorganisms. The tested apple pomace oligosaccharides did not stimulate the adherence of all beneficial bacteria. Thus, when constructing symbiotic preparations (prebiotic plus probiotic), it is important to select beneficial bacteria that provide colonization of the gut and are additionally stimulated by the chosen prebiotic. At the same time, the prebiotic apple pomace did not inhibit adherence of all the tested pathogenic strains, as expected. However, it is worth mentioning that probiotic bacteria can also demonstrate antagonistic activity towards gut pathogens, counteracting their colonization, so they should be selected based on these two features.

### 2.4. Antiradical Activity

Based on the results obtained for individual oligosaccharide fractions, an increase in antioxidant activity was observed, along with a decrease in their molecular weight (Table 2). The oligosaccharide fraction with an MW below 10 kDa showed the highest anti-radical activity. In this fraction, saccharides with a mass of 100 Da–1 kDa (7.47 and 1.31 mM Trolox/100 mg relative to the ABTS and DPPH radicals) were characterized by the greatest ability to scavenge free radicals.

### 2.5. PCA Analysis

**Figure 10:**
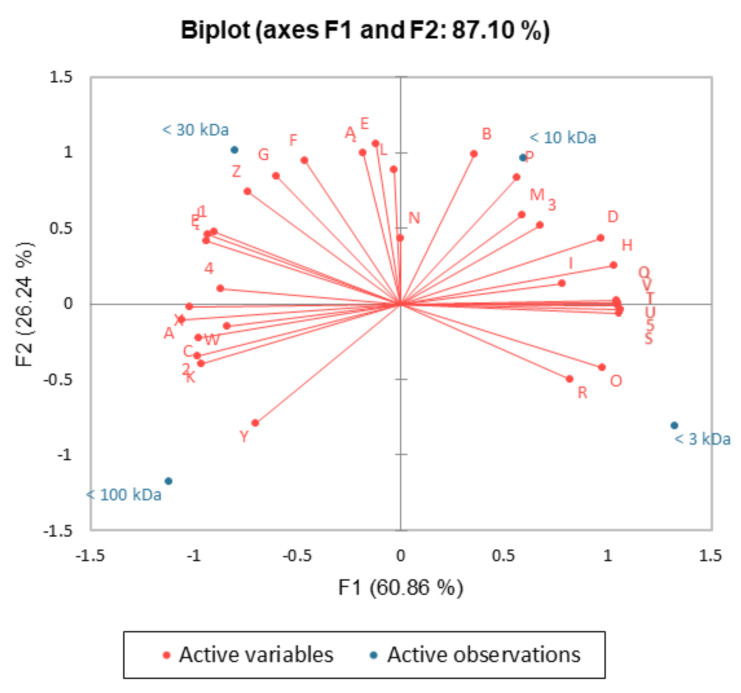
PCA biplot showing the relationships between the molecular weight, prebiotic activity, and chemical composition of POS, with adherence to collagen (A—*E. coli* ATCC 10536, B—*S. typhimurium* ATCC 14028, C—*E. facealis* ATCC 29212, D—*L. monocytogenes* ATCC 19115, E—*L. plantarum* 0981, F—*L. brevis* 0984, G—*Bifidobacterium* ssp. 76/1/1, H—*Bifidobacterium* ssp. 71/1/2), adherence to mucous (I—*E. coli* ATCC 10536, J—*S*. Typhimurium ATCC 14028, K—*E. facealis* ATCC 29212, L—*L. monocytogenes* ATCC 19115, M—*L. plantarum* 0981, N—*L. brevis* 0984, O—*Bifidobacterium* ssp. 76/1/1, P—*Bifidobacterium* ssp. 71/1/2), POS fermentability (Q—*E. coli* ATCC 10536, R—*E. coli* ATCC 2739, S—*S. typhimurium* ATCC 14028, T—*S. typhimurium* ATCC 13311, U—*L. monocytogenes* ATCC 19115, V—*L. monocytogenes* ATCC 195, W—*L. plantarum* 0995, X—*L. plantarum* 0989, Y—*L. brevis* 0984, Z—*Bifidobacterium* ssp. 1, Ą—*Bifidobacterium* ssp. 2, Ę—*Bifidobacterium* ssp. 3), and chemical composition (1—xylose, 2—rhamnose, 3—galactose, 4—arabinose, 5—galacturonic acid).

A PCA test was performed to reveal the relationship between the molecular weight of the POSs and their fermentability, as well as their simulation/inhibition of adherence and composition. By analyzing the percentages of variance, it was observed that 87.10% of the total variance was captured by only two components. The principal component (F1) contributed 60.86%, and the secondary component (F2) accounted for 26.24% of the variance (Figure 10). In the final part of the study, case classification was performed on the basis of the correlation for each PCA factor. The PCA analysis confirmed the differences in biological activity between the investigated oligosaccharides, as the four different POS fractions were distributed in different quadrants of the coordinate system. Factor F1 seemed to explain the distribution of molecular weights of the oligosaccharides. For POSs characterized with the lowest molecular weight, the content of galacturonic acid was correlated with their fermentability by pathogenic bacteria (*E. coli* ATCC 10536, *E. coli* ATCC 2739, *S. typhimurium* ATCC 14028, *S. typhimurium* ATCC 13311, *L. monocytogenes* ATCC 19115, *L. monocytogenes* ATCC 195). POSs with the average molecular weight (MW < 10 kDa, MW < 30 kDa) were explained by the F2 factor. This group highly correlated with the adherence to collagen mainly by beneficial microorganisms (*L. plantarum* 0981, *L. brevis* and *Bifidobacterium* ssp. 76/1/1) and pathogenic *S.* Typhimurium ATCC 14028, as well as the adherence to mucous (by *Bifidobacterium* ssp. 71/1/2, *L. monocytogenes* ATCC 19115). Based on the contribution and squared cosines of the variables, the F2 factor group also included fermentability by *L. brevis* 0984, *Bifidobacterium* ssp. 1, and *Bifidobacterium* ssp. 2.

## 3. Materials and Methods

### 3.1. Materials

Apple pomace (AP) was obtained from ZPOW Agros Nova Sp. z o.o. (Łowicz, Poland) and stored at −20 °C prior to use. The AP was hydrolyzed using commercial enzyme preparations Rohapect Ma Plus T and Rohament CL (AB Enzymes, Darmstadt, Germany).

### 3.2. POS Production

Pectin-derived oligosaccharides were generated in the process of enzymatic hydrolysis described previously by Wilkowska et al. [8]. Briefly, the AP was blended with water in proportions of 1:3 (*w*/*w*) and homogenized to obtain 0.8–1.0 mm particles. The pH of the mixture was adjusted to 4.0 using 0.1 M NaOH. The cellulolytic enzyme Rohament CL (AB Enzymes) and the pectinolytic enzyme Rohapect Ma Plus T (AB Enzymes) were used at doses of 37 ppm and 75 ppm, respectively. The samples were incubated at 40 °C for 20 min, after which the enzymes were inactivated by denaturation performed at 85 °C for 10 min.

### 3.3. POS Fractionation

Oligosaccharide fractionation was performed by ultrafiltration. The initial hydrolysate was fractionated using an Amicon Ultra-15 centrifuge ultrafiltration tube with 100 kDa, 30 kDa, and 10 kDa Ultracel^®^ cut-off ultrafiltration membranes (Merck). Three oligosaccharide fractions were obtained, with masses in the ranges of 30–100 kDa (F1 30–100 kDa), 10–30 kDa (F2 10–30 kDa), and less than 10 kDa (F3 < 10 kDa). Portions of the F3 fraction (<10 kDa) were further fractionated using high-performance size exclusion chromatography (HPSEC). Separation was carried out using the UHPLC Dionex model UltiMate 3000 system, consisting of a UV-VIS detector with a DAD 3000 diode matrix, WPS-3000TFC autosampler (Thermo Fisher Scientific Inc., Waltham, MA, USA), and columns (300 mm × 7.8 mm ID × 3/8 in OD) by Phenomenex (Torrance, CA, USA) with Bio-Gel P-2 filling.

### 3.4. NMR Analysis

Freeze-dried oligosaccharide fractions with different molecular masses were tested: F1 (30–100 kDa), F2 (10–30 kDa), and F3 (3–10 kDa). Samples of 30–50 mg were dissolved in 0.6 mL D2O (with the addition of 0.1% TSP internal standard). The solutions prepared in this way were transferred to NMR tubes. Additionally, NMR spectra for saccharides, including arabinose, rhamnose, galactose, xylose, fructose, and glucose, were recorded during the measurements. In the course of the analysis, other patterns were also used, which were taken from their own relationship database or the Internet.

Spectra for 1H, 13C NMR, COSY, HSQC, and HMBC were recorded using an ASCEND III 500 MHz (11.74 T) NMR spectrometer from Bruker. Measurements were carried out at 308 °K. The COSY spectrum allowed for the observation of spin systems coupling to each other with three chemical bonds. In addition, the TOCSA spectrum was measured at 300 °K to observe the full spin system.

### 3.5. Acid Posthydrolysis and Monosaccharide Analysis

The POS fractions were subjected to acid hydrolysis with 2 mL of 2 M TCA per 30 mg of liophilisate at 120 °C for 2.5 h. After neutralization of the samples with 1 M NaOH, the generated monosaccharides (glucose, fructose, mannose, galactose, arabinose, xylose) were determined by UV spectrophotometry using Megazyme (Wicklow, Ireland) enzymatic kit tests. The assays were performed using a Multiscan GO spectrophotometer (Thermo Scientific) equipped with a microplate reader. Glucooligosaccharides, galactooligosaccharides, xylooligosaccharides, and arabinooligosaccharides were determined on the basis of the amounts of monosaccharides produced during acid hydrolysis [32].

### 3.6. Antiradical Activity Assay

Radical scavenging activity against ABTS^+•^ (2,2′-azinobis 3-ethylbenzothiazoline-6-sulfonic acid) was determined spectrophotometrically. The radical mixture was generated by reaction of 7 mmol/L ABTS in water with 2.45 mmol/L potassium persulphate. The fractions of oligosaccharides dissolved in water (0.2 mL) were mixed with 4 mL of ABTS reagent and incubated for 30 min. Absorbance was measured at 734 nm. The results were presented in mmol of Trolox per 100 mg of oligosaccharides, using the relevant calibration curve.

Radical scavenging activity against DPPH^+•^ was also determined spectrophotometrically. The radical mixture was prepared by dissolving 2.5 mg of DPPH in methanol. Fractions of oligosaccharides dissolved in water (0.2 mL) were mixed with 4 mL of DPPH reagent and incubated for 30 min. Absorbance was measured at 515 nm. The results were presented in mmol of Trolox per 100 mg of oligosaccharides, using the relevant calibration curve [33].

### 3.7. POS Fermentability Assay

Pure bacterial cultures were acquired from the Collection of the Institute of Fermentation Technology and Microbiology (ŁOCK 105), Lodz University of Technology, Poland, abbreviated as ŁOCK. Bacterial strains were purchased from the American Type Culture Collection, abbreviated as ATCC. The following strains were used: *Lactiplantibacillus* sp. (*L. plantarum* ŁOCK 0995, *L. plantarum* ŁOCK 0981, *L. plantarum* ŁOCK 0989), *Levilactobacillus brevis* ŁOCK 0984), *Bifidobacterium* sp. (Bifidobacterium 1, Bifidobacterium 2, Bifidobacterium 3—strains isolated from human feces), and pathogens *(Escherichia coli* ATCC 10536, *Escherichia coli* ATCC 8739, *Salmonella enterica* Typhimurium ATCC 14028, *Salmonella enterica* Typhimurium ATCC 13311, *Listeria monocytogenes* ŁOCK 18195, *Listeria monocytogenes* ATCC 19115, *Enterococus faecalis* ATCC 29212, *Enterobacter cloaceae* ATCC 13047, *Clostridium difficile* ATCC 43593, *Clostridium difficile* ATCC 9689).

The analyzed strains (2% of inoculum) were seeded individually on modified glucose-free medium containing 2% of an oligosaccharide fraction or 2% of unmodified apple pectin. The strains were incubated for 48 h at 37 °C. Quantitative evaluation of microorganisms was performed by seeding on selective media: TSA Agar (Merck, Darmstadt, Germany)—*Escherichia* sp., *Enterococcus* sp., *Enterobacter* sp., *Klebsiella* sp., *Pseudomonas* sp., *Staphylococcus* sp., *Salmonella* sp., *Listeria* sp.; De Man Rogosa and Sharpe (MRS) Agar (Merck, Darmstadt, Germany)—*Lactobacillus* sp.; *Bifidobacterium* medium (Merck, Darmstadt, Germany)—*Bifidobacterium* sp.; and Agar TSC (Merck, Darmstadt, Germany)—*Clostridium* sp. Incubation was carried out for 48 h under aerobic or anaerobic conditions depending on the tested microorganism at 37 °C. After this time, the colonies were counted.

To assess the extent to which the prebiotics support selective growth of lactobacilli and bifidobacteria, the prebiotic activity index was calculated as the ratio of the number of the log CFU/mL of bacteria cultivated on the oligosaccharide containing medium to the number of the log CFU/mL of bacteria cultivated on apple pectin medium.

### 3.8. Antiadhesive Activity

#### 3.8.1. Strains of Bacteria

Two strains of *Lactobacillus* sp. bacteria were used: *Lactiplantibacillus plantarum* ŁOCK 0981 and *Levilactobacillus brevis* ŁOCK 0984, both isolated from plant silages. Additionally, two strains of *Bifidobacterium* ssp. were used, 76/1/1 and 71/1/2, isolated from baby feces. The strains were acquired from the collection of the Institute of Fermentation Technology and Microbiology (ŁOCK 105) and from the collection at the Department of Environmental Biotechnology, Łódź University of Technology, Poland. Also, the following pathogens were used: *Listeria monocytogenes* ATCC 19115, *Salmonella enterica* serovar Typhimurium ATCC 14028, *Escherichia coli* ATCC 10536, and *Enterococcus faecalis* ATCC 29212. The strains were stored in Cryobanks™ (Mast Diagnostics, Reinfeld, Germany) at −22 °C. Prior to the experiment, they were activated by threefold passages in liquid De Man Rogosa and Sharpe (MRS) broth (Merck—Millipore, Darmstadt, Germany) and nutrient broth with glucose (Merck-Millipore, Darmstadt, Germany), then incubated at 37 °C for 24 h. Stock cultures were stored for 24 h at 4–5 °C.

#### 3.8.2. Adherence Assays

Adherence assays were conducted according to the method developed in our previous studies, with modifications [34,35]. First, 24 h cultures were centrifuged (3852 pm, 15 min, 4 °C), suspended in phosphate buffer saline (PBS, pH 7.2), and centrifuged again. The final optical density of the suspensions was measured spectrophotometrically (Spectroquant Prove 300, S Merck—Millipore, Darmstadt, Germany) at 630 nm and adjusted to approximately 1.0 ± 0.1 by dilution with PBS.

For adherence to collagen, bacterial suspensions in an appropriate apple preparation were added to collagen-coated 96-well plates (Becton, Dickinson and Co., Franklin Lakes, NJ, USA), with four replicates each. The bacteria were incubated for 2 h at 37 °C, rinsed with water, fixed with 80% ethanol (15 min), and stained with 0.1% crystal violet (15 min). Next, the wells were washed out with water and then with 33% acetic acid (15 min, 120 rpm).

The adherence of bacteria to mucous was determined as follows. The 96-well plate was coated with mucous from porcine stomach (150 mg/mL in PBS; pH 7.2, Type II, Sigma-Aldrich, St. Louis, MO, USA) (72 h, 4 °C). The unattached mucous was removed and washed out with PBS. The bound mucous was fixed (20 min, 60 °C). Next, bacterial suspensions were added to the wells in four replicates each and incubated for 2 h at 37 °C. Subsequently, the non-adhered bacteria were removed and washed with PBS. The remaining bacteria were fixed (20 min at 60 °C). The adhered bacteria were stained with 0.1% crystal violet (15 min) and washed with PBS, and finally, citrate buffer (20 mMol/L; pH 4.3) was added to each well (45 min, 120 rpm) to wash out the stain from the adhered bacteria. The absorbance was measured at 630 nm using a microplate reader (TriStar2 LB 942, Berthold Technologies GmbH and Co. KG, Bad Wildbad, Germany). Control samples consisted of bacterial strains in PBS, while collagen/mucous served as the background. The mean absorbance of the background was subtracted from the measured mean absorbance for each sample. Then, the stimulation/inhibition rate [%] of adherence was calculated as follows: [adherence of the strain in the presence of apple preparation/adherence of the strain in PBS × 100] – 100. This calculation provided the percentage of the increased/reduced adhesion of bacteria in the presence of apple pomace.

### 3.9. Statistical Analysis

Statistical analyses were performed using the XLSTAT program (ADDINSOFT, Lumivero, France). Data were analyzed using two-way analysis of variance (ANOVA). The differences between samples with normal distributions were evaluated using a Tukey test. Significant differences were accepted at *p* < 0.05. Principal component analysis (PCA) with Varimax rotation was performed. A 4 × 40 matrix was constructed for the obtained oligosaccharide fractions and different aspects of their biological activity and chemical composition. The results were presented as the mean ± standard deviation (SD). All experiments were performed in triplicate.

## 4. Conclusions

In this research, we investigated the prebiotic effects of different fractions of pectin-derived oligosaccharides (POSs) from apple pomace in relation to their molecular weight (MW), structure, and composition. Controlled enzymatic degradation of apple pomace pectin resulted in POS samples with varying molecular weights. Oligosaccharides with MW < 30 kDa showed a significantly higher prebiotic score for both lactobacilli and bifidobacteria. This fraction also demonstrated the greatest biological activity towards adhesion of the tested microorganisms. Therefore, oligosaccharides with MW < 30 kDa appear to be the most promising prebiotic candidates. To our knowledge, the ability to support the adherence of beneficial or pathogenic microorganisms to collagen and mucous as a function of the molecular weight of POS fractions has not been extensively studied before. Therefore, it is regarded as a novelty of this work.

In our research, POS fermentability as well as antiadhesive activity were tested using simple in vitro models involving single strains of microorganisms. However, carbohydrate fermentation in the colon is a cooperative process, involving a different consortia of bacterial species. Further studies are planned, using a continuous model of the gastrointestinal tract. Such models enable the cultivation of the complex human intestinal microbial ecosystem under representative conditions for the different intestinal regions. These models can provide detailed information on not only the fermentation profile but also the localization of specific effects along the intestinal tract.

## Figures and Tables

**Table 1:** Percentages of glycosidic bounds (α(1–2), α(1–3), α(1–4), and α(1–6)) attributed to oligosaccharides of different molecular weights.

Molecular Weight	α(1–2) and α(1–3)	α(1–4)	α(1–6)
<10 kDa	43.5	30	16
10–30 kDa	60	24	7
30–100 kDa	61	29	1

**Table 2:** Antioxidant activity of the oligosaccharide fractions of the prebiotic preparation against the ABTS and DPPH radicals. Data represent means from three replicates in one experiment. Values denoted by different letters in the columns indicate statistically significant differences between the oligosaccharide MW samples, ANOVA (*p* < 0.05).

Molecular Weight	DPPH [mM TE/100 mg]	ABTS [mM TE/100 mg]
MW 100 Da–1 kDa	1.31 ± 0.05 e	7.47 ± 0.28 d
MW 1–3 kDa	1.02 ± 0.03 d	5.81 ± 0.22 c
MW 3–10 kDa	0.35 ± 0.01 c	3.45 ± 0.01 a
MW < 10 kDa	0.93 ± 0.03 ad	4.83 ± 0.19 b
MW 10–30 kDa	0.67 ± 0.02 b	4.33 ± 0.01 b
MW 30–100 kDa	0.81 ± 0.04 a	3.55 ± 0.16 a

## Data Availability

Additional raw data can be obtained through contact with the corresponding author.
